# In Vitro Investigation of Material Combinations for Meso- and Suprastructures in a Biomimetic Approach to Restore One-Piece Zirconia Implants

**DOI:** 10.3390/ma16041355

**Published:** 2023-02-05

**Authors:** Reto Nueesch, Sabrina Karlin, Jens Fischer, Nadja Rohr

**Affiliations:** Biomaterials and Technology, Department of Reconstructive Dentistry, University Center for Dental Medicine Basel UZB, University of Basel, 4001 Basel, Switzerland

**Keywords:** mesostructure, suprastructure, microfilled composite, feldspathic ceramic, lithium-disilicate, zirconia, cementation, cement excess, ceramic implant

## Abstract

The aim of this study was to find a suitable material combination to avoid cement excess in the marginal region of one-piece zirconia implant-supported restorations by means of a hybrid crown consisting of a meso- and a suprastructure. One-piece zirconia implants (*n* = 120) were embedded in epoxy resin. Microfilled resin composite mesostructures (*n* = 60), designed as caps, were bonded on the implant abutment with a primer only. A molar crown was constructed and cemented with a resin cement on top of the mesostructure as a suprastructure out of feldspar ceramic (*n* = 12), lithium-disilicate (*n* = 24), or zirconia (*n* = 24). Fracture load (*n* = 6) and retention force (*n* = 6) were measured immediately after storage in distilled water at 37 °C for 24 h, as well as after an additional exposure to artificial aging in a chewing simulator and simultaneous thermal cycling. For the measurement of the fracture load, monolithic crowns made of the employed restorative materials and identical in shape to the hybrid crowns served as controls (*n* = 6 each). Fracture load values for feldspar ceramic and lithium-disilicate hybrid crowns were slightly higher than those for the respective monolithic crowns at baseline and after aging, which was statistically significant only for feldspar crowns after aging. In contrast, fracture load values for zirconia monolithic crowns were higher than those for zirconia hybrid crowns, which was only statistically significant after aging. Artificial aging reduced the fracture load of feldspar and lithium-disilicate crowns both for hybrid and monolithic crowns. The effect was only statistically significant for lithium disilicate hybrid crowns. The fracture load for hybrid and monolithic zirconia crowns was increased by artificial aging without reaching statistical significance. The retention force of lithium-disilicate and zirconia hybrid crowns was not affected by artificial aging. Taking into account retention force and fracture load, lithium-disilicate hybrid crowns showed promising results.

## 1. Introduction

Due to the clinical success in restoring aesthetics and function in edentulous areas of the dentition, titanium dental implants have become the gold standard treatment option for replacing missing teeth [1]. However, clinical signs of hypersensitivity to titanium were found [2], calling into question the biocompatibility of titanium. Further, the greyish shadow of titanium may be visible through the peri-implant mucosa in patients with a thin gingival phenotype, thus affecting the aesthetic outcome [3].

With its tooth-like color, zirconia (ZrO_2_) has been introduced as a promising material for implants [4,5,6,7,8,9]. Survival rates of 94.3% to 98.4% after three to five years of functional loading with a mean peri-implant bone loss of 0.7–1.0 mm have been reported [10,11,12,13].

The majority of zirconia implants produced are one-piece implants [14,15,16]. Cementation is mandatory to fix the restorations on a one-piece implant. From a mechanical point of view, laboratory testing suggests that monolithic restorations combined with a resin composite cement with high compressive strength should be used for cementing the restoration on the implant abutment [17,18,19,20]. The reported five-year survival rates of one-piece ceramic implant-supported single crown reconstructions were 89% for veneered zirconia and 100% for monolithic lithium-disilicate ceramic [13].

To avoid the visibility of the crown margin in the aesthetic zone with its highly scalloped mucosa, the implant shoulder is often placed subgingivally. Consequently, the risk of accidentally leaving excess cement in the submucosal area increases [21,22,23]. Excess cement may lead to local infection, occasionally causing mucositis or—even worse—peri-implantitis [22,23,24,25,26,27,28]. In general, cemented restorations exhibit more biologic complications than screw-retained restorations [29]. Even crown venting, the most effective way to avoid cement excess, does not completely prevent cement residues [30]. 

The aim of the present study was to find a suitable material combination for excess-free cementation regarding fracture load and retention force by means of a biomimetic approach on one-piece zirconia implants in vitro. As previously described, the concept is based on a restoration consisting of a meso- and a suprastructure [31]. The initial idea was to bond a polymer-infiltrated ceramic crown to the abutment during implant surgery using a resin composite cement. Thus, excess cement can be removed under visual control prior to wound closure. After a healing period, the polymer-infiltrated ceramic crown is prepared intraorally, analogous to a crown preparation on a natural tooth with an epigingival preparation margin. The reduced resin composite crown now functions as a mesostructure and is scanned with an intraoral scanner. After scanning, a ceramic crown (suprastructure) is fabricated and bonded with a resin composite cement to the mesostructure. The cement excess is now removed just as the excess removal on a natural tooth on a tissue level with easy access would be removed.

However, that approach extends the surgical session, the polymer-infiltrated ceramic crown must be placed under sterile conditions, and the process is complex. With the use of a bonding agent only instead of applying a resin cement, there would be no cement excess. The bonding agent must be applied in a thin layer, only without any excess material. The cementation of the mesostructure then may be performed after wound healing. A precise fit of the mesostructure on the abutment is prerequisite. As tolerances in the production process of some micrometers must be taken into account, only negative tolerances should be adjusted in order to have a press fit. Therefore, a material with a low elastic modulus must be chosen for the mesostructure; the polymer-infiltrated ceramic is not suitable for that purpose. However, a suitable material would be CAD-Temp, a microfilled resin composite used to fabricate temporary crowns (Vita, Bad Säckingen, Germany). For bonding, Scotchbond Universal Plus Adhesive (3M, Neuss, Germany) is currently the only primer available that is dual-curing, contains filler, and is able to bond to polymer materials, silicate, and oxide ceramics [32]. Different materials might be suitable for fabricating the suprastructure, which can be cemented on the mesostructure in a traditional way with a resin composite cement.

The aim of the study was to find a suitable combination of materials for the meso- and the suprastructure regarding retention force and fracture load before and after chewing simulation.

## 2. Materials and Methods

### 2.1. Implant Preparation

One hundred twenty one-piece zirconia implants (ceramic.implant, Vita, Bad Säckingen, Germany) with a diameter of 4.0 mm and a length of 8.0 mm in the intraosseous part were embedded in epoxy resin (RenCast CW 20/Ren HY 49, Huntsman Advanced Materials, Duxford, UK) according to ISO standard 14801 [33]. Following the standard to simulate alveolar bone resorption, a 3 mm clearance between the implant neck and the epoxy surface was maintained. To ensure fixation in the chewing simulator and the universal testing device, all specimens were embedded in specially manufactured cylindrical silicone molds (internal dimensions: diameter: 16 mm, height: 35 mm).

### 2.2. Production of the Restorations

**Figure 1:**
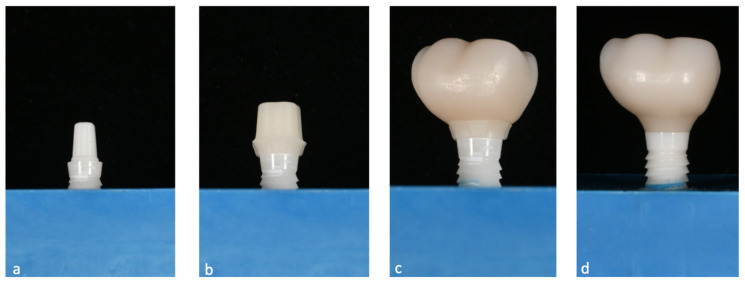
(**a**) One-piece ceramic implant; (**b**) Mesostructure on the implant; (**c**) Suprastructure on mesostructure on the implant; (**d**) Monolithic crown on the implant.

The design of the mesostructures, suprastructures, and monolithic crowns has been previously described [31] and is shown in Figure 1. 

By means of high-precision machining (Ultrasonic 20 linear, DMG MORI, Tokyo, Japan), 60 mesostructures identical in shape were ground from a microfilled resin composite consisting of 14 wt% inorganic filler and 86 wt% acrylate polymer (Vita CAD-Temp, Vita).

Suprastructures were manufactured from feldspar ceramic (Vitablocs Mark II, Vita; *n* = 12), lithium-disilicate ceramic (IPS e.max CAD, Ivoclar Vivadent, Schaan, Liechtenstein; *n* = 24), and zirconia (Vita YZ ST, Vita; *n* = 24) with the milling devices specified in Table 1.

A monolithic full-contour restoration was designed by merging the meso- and suprastructure without geometric changes (Meshmixer, V3.5.474, Autodesk, San Rafael, CA, USA). These restorations were milled as described in Table 1 and served as controls. Twenty-four monolithic restorations of microfilled resin composite (Vita CAD-Temp, Vita) were used to measure fracture load (baseline *n* = 6 and aged *n* = 6) and retention force (baseline *n* = 6 and aged *n* = 6) before and after artificial aging. Twelve monolithic restorations each of feldspar ceramic (Vitablocs Mark II, Vita), lithium-disilicate ceramic (IPS e.max CAD, Ivoclar Vivadent), and zirconia (Vita YZ ST, Vita) were used to measure fracture load values before and after artificial aging.

Prior to polishing the occlusal surface of all specimens according to the manufacturers’ specifications, the lithium-disilicate was crystallized, and zirconia was sintered in a dental furnace (Programat CS2, Ivoclar Vivadent/ VITA Zircomat 6100, Vita) according to the manufacturers’ instructions.

### 2.3. Cementation of the Restorations

All meso- and suprastructures, as well as the monolithic restorations, were cleaned in an ultrasonic bath (TPC-15, Telsonic AG, Bronschhofen, Switzerland) with ethanol for 60 s. The abutment portions of the implants were cleaned with a foam pellet soaked in ethanol and air dried with oil-free air.

The intaglio surfaces of the suprastructures and monolithic restorations made of feldspar ceramic and lithium-disilicate ceramic were acid etched for 60 s and 20 s [34], respectively, with 5% hydrofluoric acid (Vita Adiva Etch, Vita), cleaned with oil-free water-spray, ultrasonically rinsed in ethanol (TPC-15), and dried with oil-free air. The intaglio surface of the zirconia specimens underwent air-particle abrasion (Al_2_O_3_, 50 μm, 5 s) and ultrasonic cleaning in ethanol (TPC-15).

**Figure 2:**
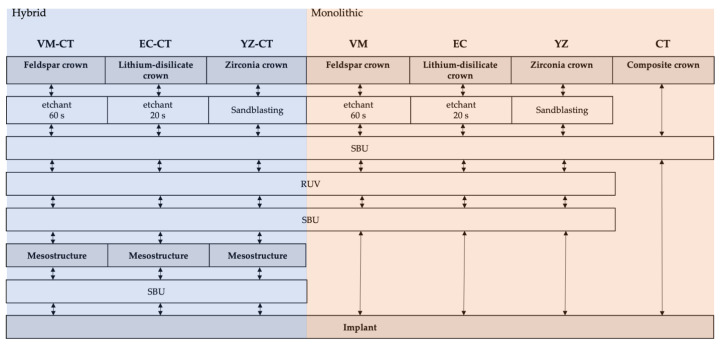
Test matrix describing the cementation procedure of mesostructures on implants and suprastructures on mesostructures (Hybrid), as well as the cementation of monolithic crowns on implants (Monolithic).

Pretreatment of abutments and meso- and suprastructures, as well as cementation of the monolithic restorations, was performed using an adhesive resin composite cement system (RelyX Universal, RUV, Table 2) according to the test matrix presented in Figure 2 and following the manufacturer’s instructions. 

Between implant and mesostructure, only the primer (3M Scotchbond Universal Plus Adhesive, 3M [SBU]) was applied to avoid cement excess. After applying the primer, the mesostructures were placed on the abutment with finger pressure, followed by light-polymerization for 20 s from each side (Elipar DeepCure-S, 3M). Suprastructures were cemented on the mesostructures, applying the primer and the resin composite cement (“hybrid crowns”). Excess cement was removed with foam pellets. The procedure for cementing the monolithic restorations was the same as for the suprastrucures, except for the monolithic resin composite restoration (CT) where the primer only was used. All specimens were placed in an alignment apparatus at room temperature, and a force of 20 N was applied for 15 min. After cementation, the specimens were stored in deionized water at 37 °C for 24 h (WTC binder, Binder, Tuttlingen, Germany).

### 2.4. Artificial Aging

After storage in water for 24 h, specimens of each group were loaded in sets of 12 in a custom-built chewing simulator parallel to the implant axis in the central fissure with a zirconia ball antagonist (diameter: 5 mm) for 1.2 million cycles using a load of 49 N and a frequency of 1.5 Hz to simulate aging of five years clinical function [35,36]. Simultaneously, the specimens underwent thermal cycling for 6000 cycles between 5 and 55 °C with a 120 s dwell time and a 5 s transfer time.

The fracture load and retention force of all groups were measured immediately after water storage (“baseline”) and after artificial aging (“aged”). The number of test specimens in each group was *n* = 6.

### 2.5. Fracture Load

To measure the fracture load, specimens were loaded parallel to the implant axis in the central fissure with a steel ball (diameter: 4.76 mm). To avoid force peaks, a 0.2 mm thick tin foil (Dentaurum, Pforzheim, Germany) was placed between the steel ball and the occlusal surface of the restoration. All specimens were successively placed in the universal testing machine (Z020, Zwick/Roell, Ulm, Germany), and a force was applied until fracture with a crosshead speed of 1 mm/min. After fracture, the implants were inspected for cement residues with dental loupes (Swarovski, Starmed, Munich, Germany) at a magnification of 4.5×.

### 2.6. Retention Force

To measure the retention of the restorations on the abutments, all specimens were successively placed in the universal testing machine (Z020) in a customized specimen mount which was lined with a molded Teflon inlay to avoid force peaks. A tensile force was applied with a crosshead speed of 1 mm/min, and force at debonding of the restoration was recorded. The implants were inspected for cement residues with dental loupes (Swarovski) at a magnification of 4.5×. Additionally, the location of retention loss was recorded (between implant and mesostructure or mesostructure and suprastructure).

### 2.7. Flexural Strength of Restorative Materials

The flexural strength of the restorative materials CT, VM, EC, and YZ was measured before and after aging with a 3-point flexural test (*n* = 20). Bending bars (1.2 mm × 4.0 mm × 20.0 mm) were fabricated from the CAD/CAM blocks of each restorative material (CT, VM, EC) using a diamond wire saw (Precision Diamond Wire Saw Well 3242, WELL Diamond Wire Saws SA, Le Locle, Switzerland). The surfaces were polished using silicon carbide paper grit 1200. The bending bars made of YZ were drawn in Meshmixer (MeshAutodesk) and milled from the same blank as the restorations were previously. Edges of YZ bars were chamfered using silicon carbide paper grit 1200. Bending bars of EC were crystallized, and YZ bars were sintered as described above. Flexural strength was measured at baseline (storing in water at 37 °C for 24 h (WTC binder, Binder)) and after aging. Aged specimens additionally underwent thermocycle aging, being subjected to 6000 cycles between 5 and 55 °C with a dwell time of 30 s (Thermocycler THE-1100, SD Mechatronik GmbH, Feldkirchen, Germany).

The bending bars were placed in a universal testing machine (Z020 Zwick/Roell) and loaded to failure at a crosshead speed of 0.5 mm/min. The bending strength *σ* was calculated:

$$\sigma =\frac{3Fl}{2wh^{2}}$$

*F* is the fracture load; *l* is the roller spacing (15 mm); *w* is the width and *h* is the height of the bar. 

### 2.8. Statistical Analysis

A sample size of *n* = 6 for fracture load and retention force testing was chosen based on the outcome of a previous study using a similar test set-up [31]. Within each material group, two-way ANOVAs were performed to test for the effects of aging and construction followed by Fisher’s least significant difference (LSD) post-hoc tests. For groups with the same aging protocol and the same design, one-way ANOVAs were carried out to test for differences between the materials using Fisher’s LSD post-hoc tests (Stat Plus Pro V.6.1.25, Analyst Soft, Walnut, CA, USA). The level of significance was set to 0.05. Results were presented descriptively using mean ± standard deviation.

## 3. Results

After artificial aging, no damage was detected on any of the restorations. Therefore, data of all 120 specimens could be applied for the evaluation. All *p*-values are listed in the Appendix A (Table A1 and Table A2).

### 3.1. Fracture Load

Eighteen out of 84 implants fractured during the fracture load test (Table 3). Fourteen of the implant fractures occurred during testing at baseline.

**Figure 3:**
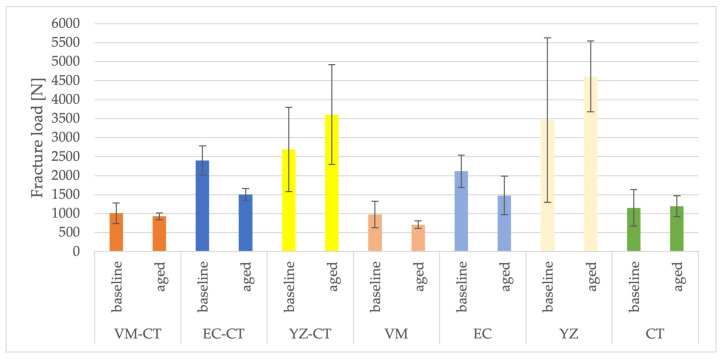
Fracture load values of the restorations (*n* = 6 per group).

Two-way ANOVA showed no statistically significant effect of aging and type of construction. Within the same materials, no significant effect of the mesostructure on the fracture load values could be found (Table 4 and Table A1, Figure 3). 

At baseline, fracture load values for VM-CT (1010 ± 274 N) were significantly lower than those for EC-CT (2475 ± 374 N) and YZ-CT (2692 ± 1111 N) which did not differ significantly from each other (Table 4 and Table A1, Figure 3). After artificial aging, the fracture load values of YZ-CT (3607 ± 1311 N) were statistically significantly higher than those of VM-CT (930 ± 92 N) and EC-CT (1503 ± 159 N), which did not differ significantly from each other (Table 4 and Table A1, Figure 3). The decrease in fracture load in groups VM-CT and EC-CT after artificial aging was statistically significant for EC-CT only. The increase in fracture load values of YZ-CT after artificial aging was not statistically significant (Table 4 and Table A1, Figure 3). 

At baseline, fracture load values of the monolithic restorations were highest for YZ (3465 ± 2166 N), achieving a statistically significant difference only compared to VM (977 ± 350 N) and CT (1154 ± 479 N). The difference between VM and CT was not statistically significant. After artificial aging, the fracture load values for YZ (4613 ± 936 N) were significantly higher than those for VM (710 ± 104 N), EC (1479 ± 504 N), and CT (1246 ± 164 N). The difference between VM and EC was significant, whereas the differences between CT and VM, as well as EC and CT, were not (Table 4 and Table A1, Figure 3).

At baseline, fracture load values of hybrid restorations compared to monolithic restorations did not differ statistically significantly. After aging, EC-CT and EC did not show statistically significant differences, whereas monolithic VM showed statistically significant lower values than the hybrid crowns. In contrast, the values for monolithic YZ were statistically significant, higher than the respective hybrid version YZ-CT.

The fracture load test of VM-CT resulted in a total destruction of the suprastructure, forming 2–4 fragments, without any damage of the underlining mesostructure at baseline and after aging. For groups EC-CT and YZ-CT, aging revealed a distinct shift from fractures of the mesostructure to fractures of the suprastructure. In the monolithic groups (VM, EC, YZ, CT), all test specimens were destroyed, forming 2–5 fragments. For all specimens, the cement remained on the restorative material fragments after fracture (Table 3).

### 3.2. Flexural Strength

The flexural strength values are displayed in Table 5. Overall, a significant effect was found between materials and aging. At baseline, significantly highest values were found for YZ (840 ± 159 MPa) and lowest for CT (101 ± 6 MPa) and VM (101 ± 14 MPa) when compared to EC (378 ± 68 MPa). Values of CT and VM did not differ significantly. After aging, the flexural strength values differed significantly between all materials.

### 3.3. Correlation between Fracture Load and Flexural Strength 

**Figure 4:**
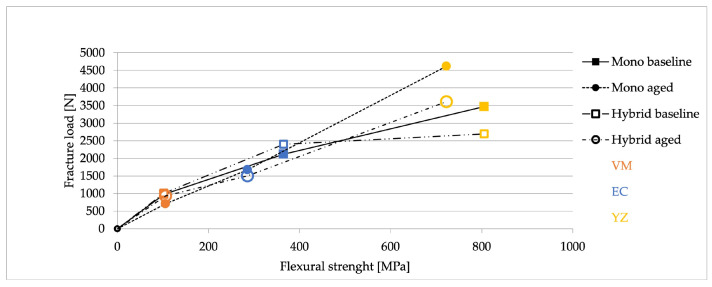
Correlation between fracture load of the specimens and flexural strength of the corresponding suprastructure and monolithic crown materials.

The fracture load values of monolithic and hybrid crowns of VM, EC, and YZ before and after aging compared with the corresponding flexural strength values are visualized in Figure 4. At baseline, both for the monolithic crowns and for the hybrid restorations, a strong non-linear correlation with a flattening curve was found. After aging, the monolithic YZ crowns showed higher strength, leading to a discontinuity in the direction of the curve.

### 3.4. Retention Force

**Figure 5:**
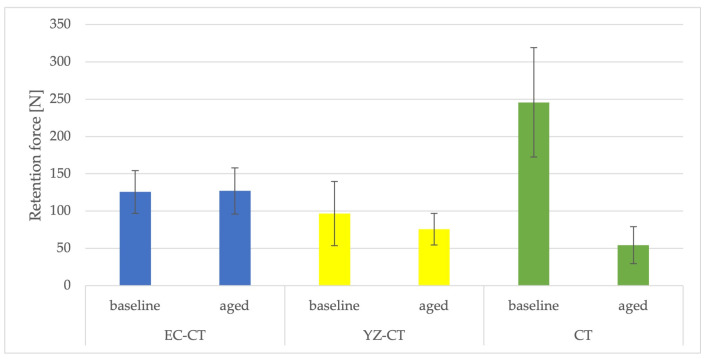
Retention force of the restorations (*n* = 6 per group).

Retention force was measured with hybrid crowns EC-CT and YZ-CT, as well as monolithic crowns CT only. In general, aging significantly affected the retention force for all material groups. At baseline, retention force values of hybrid crowns EC-CT (126 ± 29 N) and YZ-CT (97 ± 43 N) did not differ significantly from each other (Table 6 and Table A2, Figure 5). Aging resulted in reduced retention force values for YZ-CT (76 ± 21 N). EC-CT (127 ± 31 N) did not show any change in retention force after artificial aging (Table 6 and Table A2, Figure 5). At baseline, retention force of CT was significantly higher than those for EC-CT and YZ-CT. After artificial aging, retention force of CT was significantly reduced.

The failure modes of the hybrid and the monolithic restorations are shown in Table 7. No cement residues were found on the outer surface of the mesostructures or on the implant abutments, meaning that the cement stuck to the intaglio surfaces of either the mesostructure, the suprastructure, or the monolithic crown.

## 4. Discussion

The aim of the present study was to find a suitable material combination for excess-free cementation on one-piece zirconia implants by assessing fracture load and retention force of implant-supported restorations in vitro. The results suggest to further pursue the basic idea to use CT as material for the mesostructure and SBU as bonding agent.

All suprastructures and monolithic restorations in the present investigation were designed by a CAD/CAM technician; therefore, the shaping can be considered equivalent to clinically used restorations in dental practice. Prefabricated mesostructures with identical outer surface and uniform circular shoulder were used to standardize the in-vitro process. To make the manufacturing process of suprastructures close to a chairside approach, suprastructures were milled deploying milling units commonly used in dental practice.

It is well known that bonding to zirconia is increased by air-particle abrasion in order to enhance its surface area and surface energy [37]. Due to traumatization of the surrounding structures intraorally, pre-treatment of the implant abutment by sandblasting with Al_2_O_3_ was omitted in this study to mimic a clinical situation. As primers containing methacrylates with phosphate groups such as 10-methacryloyloxydecyl dihydrogen phosphate (MDP) are known to strengthen the long-term bond to zirconia, together with the high adhesion to CT [34], the RUV system was chosen for its simplicity of use and the presence of MDP-equivalence in the primer SBU [32,37,38]. CT was chosen as material for the mesostructure due to its low elastic modulus, its easy machinability, a good bonding performance with SBU [31], and the fact that it has been on the market as a material for temporary crowns for decades. Materials for the suprastructures were chosen for their differing mechanical properties.

Hybrid and monolithic restorations of the same material group did not differ in fracture load. Therefore, it can be concluded that the CT mesostructure did not affect the mechanical strength of the restoration. Facture load values after artificial aging behaved differently in the hybrid restoration groups. While VM-CT demonstrated only a slight decrease without any statistically significant difference, EC-CT showed a statistically significant decrease, and YZ-CT, in contrast, showed a statistically significant increase in fracture load. With the monolithic restorations, a similar behavior was observed. Monolithic CT was not affected by artificial aging. The increase of fracture load after artificial aging for YZ-CT and YZ may be attributed to a partial phase transition from tetragonal to monoclinic phase, which, due to the transformation toughening, increases the flexural strength of the material [39,40].

The fracture load of the monolithic restorations made of feldspar ceramics amounted to 977 ± 350 N. These values are within the range of earlier reports (951 ± 172 N) using a composite resin cement with a high compressive strength [29]. It may be concluded, therefore, that the results obtained with this test method are reproducible. When the fracture load values of the feldspar hybrid group VM-CT (baseline: 1010 ± 274 N, aged: 930 ± 92 N) are compared with the feldspar hybrid group with a polymer-infiltrated ceramic mesostructure (VITA Enamic, VITA) from an earlier study, the results are in the same range before (1148 ± 270 N) and after aging (897 ± 106 N) [31]. When comparing fracture load values of the monolithic crowns with the findings of Rohr et al. (2019), it is noticeable that significantly higher values for CT (1719 N baseline, 1409 N aged) and YZ (1719 N baseline, 6055 N aged) were obtained [41]. For the CT specimens it must be considered that, in the present study, a tight fit between abutment and intaglio surface of the crown was adjusted in order to allow a minimum space for the primer. Premature contacts are not compensated by a cement layer, and therefore might lead to local stress peaks and reduction in overall strength. For the zirconia specimens, values were higher because a high strength 3Y-TZP (3 mol%-yttria tetragonal zirconia polycrystal) was used by Rohr et al. (2019) and a 5Y-PSZ (5 mol%-yttria partially stabilized zirconia) in the present study [41]. Further, the implant diameter in that study was 4.5 mm and in the presented one 4.0 mm. A smaller implant abutment may thus have acted as stress peak initiator. Based on these results, a monolithic CT crown seems to be a viable solution for a temporary restoration.

A strong correlation could be found between fracture load values and the respective flexural strength of the restorative material at baseline. For VM-CT, EC-CT, VM, and EC, that finding also applies for the values obtained after artificial aging. However, YZ-CT, and YZ showed a strong increase in fracture load after artificial aging, a result not observed with the flexural strength specimens. The aging procedure of the restorations was a simultaneous treatment comprising chewing simulation and thermal cycling, while the bending test specimens were only thermal cycled. It can be concluded that, for the ceramics VM and EC, aging mainly is due to stress caused by thermal cycling, while YZ needs a mechanical component to simulate the aging process. The phase transition of YZ obviously is not triggered by thermal stress but mainly by mechanical stress. For future investigations, it should be kept in mind that for YZ a mechanical impact is necessary to simulate aging, while for silicate ceramics thermal stress might be sufficient.

All groups previously exceeded the maximal bite force experimentally with a bite force magnitude of 618 N [42]. However, in extreme clinical situations, force peaks of up to 1200 N in the molar region may be reached [43]. VM-CT, therefore, must be excluded as a reliable solution, especially considering that clinically more delicate constructions may also be required.

Retention force was only evaluated for hybrid restorations EC-CT and YZ-CT which provide sufficient strength for clinical application. For these material combinations, it is obvious that the bond strength of the mesostructure to the implant abutment at baseline is higher than that between meso- and suprastructure. The retention force of the monolithic CT crown is considerably higher, corresponding to the fact that for EC-CT and YZ-CT the mesostructure predominantly remains on the abutment.

Artificial aging did not affect the retention force values on EC-CT and YZ-CT. It is possible that suprastructures with a higher elastic modulus protect the underlying mesostructure and that stresses were therefore transferred more evenly.

After artificial aging, almost all hybrid specimens detached between mesostructure and implant abutment. The retention force values indicate a reduction in bond strength following artificial aging for all of the hybrid and the monolithic CT specimens. It may be concluded that artificial aging mainly affects the bond of SBU between CT and the implant abutment because monolithic CT restorations revealed a strong decrease in bond strength after artificial aging. At the interface CT/implant abutment, no surface treatments were performed, implying that both surfaces are smooth. A surface roughening on the CT side must be considered in subsequent investigations. With polymer-infiltrated ceramic mesostructures, similar retention force values were measured in the range of 100–130 N, dropping to 65–107 N after artificial aging [30]. Since a relatively new resin cement system (RUV) was chosen, comparison with other studies that have conducted retention force tests on zirconia implants is difficult. In contrast to the present findings, previously no effect of artificial aging on the bond strength of SBU to CT was observed [32]. However, in that investigation only thermal cycling was applied, whereas in the present study an additional mechanical stress by chewing simulation was employed. The low elastic modulus of CT might allow deformations under mechanical stress, leading to a higher stress at the restoration/implant abutment interface. This assumption, however, is contradicted by the fact that the retention force of the hybrid crowns also falls to that level.

The study was conducted on one-piece zirconia implants, as these currently hold the predominant position among ceramic implants. Due to the wider range of technical and biological complications, intense research is currently being conducted on two-piece ceramic implants [1,40,44,45]. However, the presented solution for an excess-free cementing process offers the option to use one-piece zirconia implants in well-managed clinical situations. The CT mesostructure seems to be applicable in regard to retention force and fracture load. The fracture analysis showed that, when the hybrid crowns were used, after aging most fractures occurred within the superstructures, and the mesostructure did not suffer any obvious damage. Therefore, in case of a technical complication with fracture of the suprastructure, the mesostructure can easily be cleaned and a new suprastructure fabricated and cemented. Removal of the excess cement at implant shoulder level is thus no longer necessary, and biological complications can be prevented. In view of the present results, a specific suprastructure material cannot be recommended. EC seems to be a good compromise, considering that the retention force of EC-CT was not affected by artificial aging and the fracture load was in an acceptable range. This statement is supported by excellent clinical results of monolithic EC restorations with a survival rate of 100% after five years [13]. EC is an easy-to-process material to be used chairside, but it also exists in a pressable version covering the lab-generated restoration path. 

A limiting factor of this study is that it is an in-vitro study which simulates the clinical situation with respect to technical complications rather to biological. To prevent peri-implantitis, regular dental hygiene and maintenance is essential. The influence of different prophylaxis instruments on monolithic single-tooth crowns and veneering ceramics is known [46,47]. Especially for CT, the effect of mechanical debridement on the integrity of the material has to be verified prior to any clinical study. Further studies on intraoral handling and maintenance of implant-supported hybrid crowns and to optimize the system, therefore, are necessary.

## 5. Conclusions

Based on the present in vitro data, which do not fully represent the clinical situation, the following can be concluded:

- EC-CT seems to be a reasonable material combination for hybrid crowns.
- A clinical study is recommended to optimize the system.
- During the healing phase of the implant, CT can be recommended as a provisional restoration.

## Figures and Tables

**Table 1:** Restorative materials and corresponding milling devices used. Elastic modulus according to manufacturer.

Materials	Abbr.	Specification	E-Modulus (GPa)	Manufacturer	Milling Device	Manufacturer
Vitablocs Mark II	VM	Feldspar ceramic	45.0	Vita	Cerec MCXL	Dentsply Sirona
IPS e.max CAD	EC	Lithium-disilicate ceramic	95.0	Ivoclar	Cerec MXCL	Dentsply Sirona
Vita YZ ST	YZ	4Y-PSZ	210.0	Vita	Programill PM7	Ivoclar
Vita CAD-Temp	CT	Microfilled composite	2.8	Vita	Ultrasonic 20 linear	DMG MORI

**Table 2:** Resin composite cement and primer used (material compositions according to the manufacturers’ product specifications).

Name	Code	Composition
VITA Adiva Etch	etchant	Hydrofluoric acid 5%
3M Scotchbond Universal Plus Adhesive	SBU	2-Propenoic acid, 2-methyl-, diesters with 4,6-dibromo- 1,3-benzenediol 2-(2-hydroxyethoxy)ethyl 3- hydroxypropyl diethers, 2-Propenoic acid, 2-methyl-, reaction products with 1,10- decanediol and phosphorus oxide, 2-Propenoic acid, 2-methyl-, 3-(triethoxysilyl)propyl ester, reaction products with silica and 3-(triethoxysilyl)- 1-propanamine, ethanol, water, synthetic amorphous silica, fumed, crystalline-free,
3M RelyX Universal	RUV	Triethylene Glycol Dimethacrylate, 2-Propenoic acid, 2-methyl-, 3-(trimethoxysilyl)propyl ester, 7,7,9(or 7,9,9)-Trimethyl-4,13-dioxo-3,14-dioxa-5,12-diazahexadecane-1,16-diyl bismethacrylate, 2-Propenoic acid, 2-methyl-, 1,1′-[1-(hydroxymethyl)-1,2-ethanediyl] ester, phosphorus oxide, Silane, trimethoxyoctyl-, hydrolysis products with silica, t-Amyl Hydroperoxide, 2,6-Di-tert-butyl-p-cresol, 2-hydroxyethyl methacrylate, Methyl Methacrylate, Acetic acid, copper(2+) salt, monohydrate

**Table 3:** Fracture type of the specimens.

Groups		Implant fracture 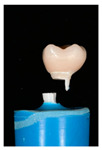	Fracture of the suprastructure 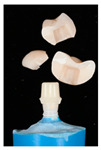	Fracture of the mesostructure 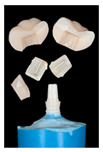	Groups	Implant fracture 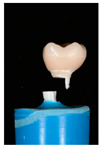	Fracture of the monolithic crown 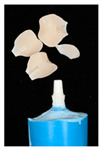
baseline	VM-CT	2	4	0	VM	0	6
aged		0	6	0		0	6
baseline	EC-CT	1	0	5	EC	1	5
aged		0	5	1		1	5
baseline	YZ-CT	3	0	3	YZ	6	0
aged		1	5	0		1	5
baseline					CT	1	5
aged						1	5

**Table 4:** Fracture load means and standard deviations. Statistically significant differences between groups determined with the Fisher-LSD post-hoc test are indicated with varying superscript letters (*p* > 0.05) (upper case horizontal, lower case vertical).

Groups	Baseline (N)	Aged (N)
VM-CT	1010 ± 274 ^A, a^	930 ± 92 ^A, a^
EC-CT	2475 ± 374 ^A, b^	1503 ± 159 ^B, a^
YZ-CT	2692 ± 1111 ^A, b^	3607 ± 1311 ^A, b^
VM	977 ± 350 ^A, a^	710 ± 104 ^A, c^
EC	2183 ± 215 ^A, b^	1479 ± 504 ^B, a^
YZ	3465 ± 2166 ^A, b^	4613 ± 936 ^A, d^
CT	1154 ± 479. ^A, a^	1246 ± 164 ^A, a, c^

**Table 5:** Flexural strength mean values and standard deviations. Statistically significant differences between groups determined with Fisher-LSD post-hoc test indicated with varying superscript letters (*p* > 0.05) (upper case horizontal, lower case vertical).

Material	Baseline (MPa)	Aged (MPa)
CT	101 ± 6 ^A, a^	78 ± 5 ^B, a^
VM	101 ± 14 ^A, a^	105 ± 10 ^A, b^
EC	378 ± 67 ^A, b^	285 ± 63 ^A, c^
YZ	841 ± 159 ^A, c^	697 ± 133 ^A, d^

**Table 6:** Retention force mean values and standard deviations. Statistically significant differences between groups determined with Fisher-LSD post-hoc test indicated with varying superscript letters (*p* > 0.05) (upper case horizontal, lower case vertical).

Groups	Baseline (N)	Aged (N)
EC-CT	126 ± 29 ^A, a^	127 ± 31 ^A, b^
YZ-CT	97 ± 43 ^A, a^	76 ± 21 ^A, a^
CT	246 ± 73 ^A, b^	54 ± 25 ^B, a^

**Table 7:** Failure mode of the specimens under retention force testing.

Groups		detachment between mesostructure and suprastructure 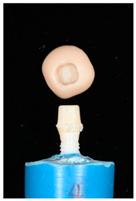	detachment between mesostructure and implant 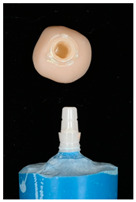	complete detachment between monolithic crown and implant 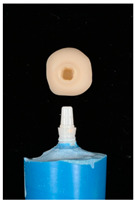
baseline	EC-CT	5	1	
aged		1	5	
baseline	YZ-CT	6	0	
aged		1	5	
baseline	CT			6
aged				6

## Data Availability

The data presented in this study are available on request from the corresponding author.

## Appendix A

**Table A1:** Fracture load: *p*-values (colored: significant difference *p* < 0.05).

Fracture Load: Hybrid															
Groups				VM-CT		EC-CT			EC-CT		YZ-CT		YZ-CT		
				aged		baseline			aged		baseline		aged		
VM-CT		baseline		0.568		<0.001					<0.001				
VM-CT		aged							0.212				<0.001		
EC-CT		baseline							<0.001		0.596				
EC-CT		aged											<0.001		
YZ-CT		baseline											0.290		
**Fracture Load: Monolithic**															
Groups			VM		EC		EC	YZ		YZ		CT		CT	
			aged		baseline		aged	baseline		aged		baseline		aged	
VM	baseline		0.079		0.077			<0.001				0.789			
VM	aged						0.022			<0.001				0.126	
EC	baseline						0.004	0.062				0.128			
EC	aged									<0.001				0.372	
YZ	baseline									0.188		0.002			
YZ	aged													<0.001	
CT	baseline													0.664	

**Table A2:** Retention force: *p*-values (colored: significant difference *p* < 0.05).

Groups		EC-CT	YZ-CT	YZ-CT	CT	CT
		aged	baseline	aged	baseline	aged
EC-CT	baseline	0.951	0.160		<0.001	
EC-CT	aged			0.004		<0.001
YZ-CT	baseline			0.463	<0.001	
YZ-CT	aged					0.226
CT	baseline					<0.001
